# Supplementary material for: Isolation and Identification of Bioactive Compounds from Streptomyces actinomycinicus PJ85 and Their In Vitro Antimicrobial Activities against Methicillin-Resistant Staphylococcus aureus
Source: Antibiotics (Basel). 2022 Dec 10;11(12):1797. doi: 10.3390/antibiotics11121797 (PMC9774200; doi:10.3390/antibiotics11121797)
Supplement: Supplementary file 1 [file antibiotics-11-01797-s001.zip › antibiotics-1996156-supplementary.pdf]

Supplementary Materials

Isolation and Identification of Bioactive Compounds from *Streptomyces actinomycinicus* PJ85 and Their In Vitro Antimicrobial Activities against Methicillin-Resistant *Staphylococcus aureus*

Panjamaphon Chanthasena [1], Yanling Hua [2], A'liyatur Rosyidah [3], Wasu Pathom-Aree [4,5], Wanwisa Limphirat [6] and Nawarat Nantapong [1,*]

[1] School of Preclinical Sciences, Institute of Science, Suranaree University of Technology, Nakhon Ratchasima 30000, Thailand
[2] The Center for Scientific and Technological Equipment, Suranaree University of Technology, Nakhon Ratchasima 30000, Thailand
[3] Research Center for Vaccine and Drug, National Research and Innovation Agency (BRIN), Bogor 16911, Indonesia
[4] Department of Biology, Faculty of Science, Chiang Mai University, Chiang Mai 50200, Thailand
[5] Center of Excellence in Bioresources for Agriculture, Industry and Medicine, Chiang Mai University, Chiang Mai 50200, Thailand
[6] Synchrotron Light Research Institute, 111 University Avenue, Nakhon Ratchasima 30000, Thailand
* Correspondence: nawarat@sut.ac.th; Tel.: +66-442-242-82

Contents

Figure S1. Colony morphology of *Streptomyces* sp. PJ85 grown on ISP-2 agar medium after incubation at 37 °C for 14 days.

Figure S2. Liquid chromatography-mass spectrometry (LC-MS) analysis of compound 1. MS spectrum with the retention time 43.9 showing the ion clusters for $[M+H]^+$ at *m/z* 1255.6466 correlated to actinomycin D.

Table S1. Comparative analysis of housekeeping genes in *Streptomyces* sp. PJ85 and *S. actinomycinicus* RCU-197^T.

Figure S1. Colony morphology of *Streptomyces* sp. PJ85 grown on ISP-2 agar medium after incubation at 37 °C for 14 days.

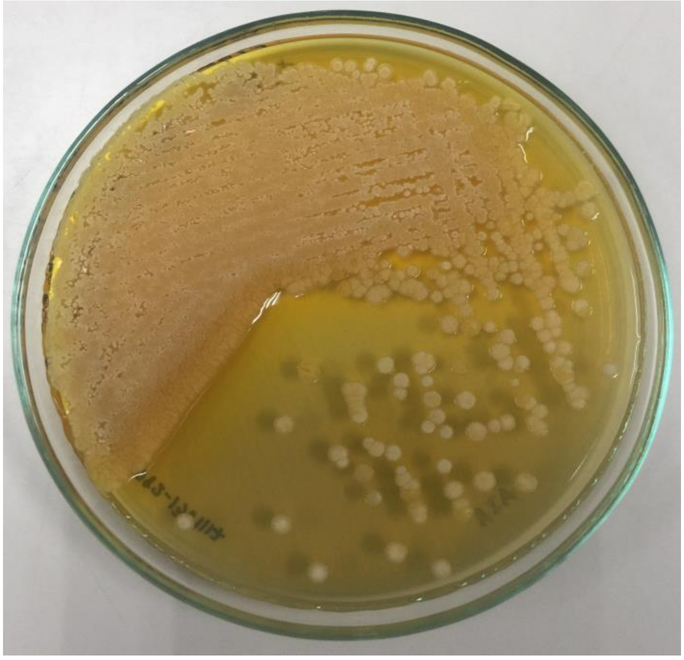

Figure S2. Liquid chromatography-mass spectrometry (LC-MS) analysis of compound 1. MS spectrum with the retention time 43.9 showing the ion clusters for $[M+H]^+$ at *m/z* 1255.6466 correlated to actinomycin D.

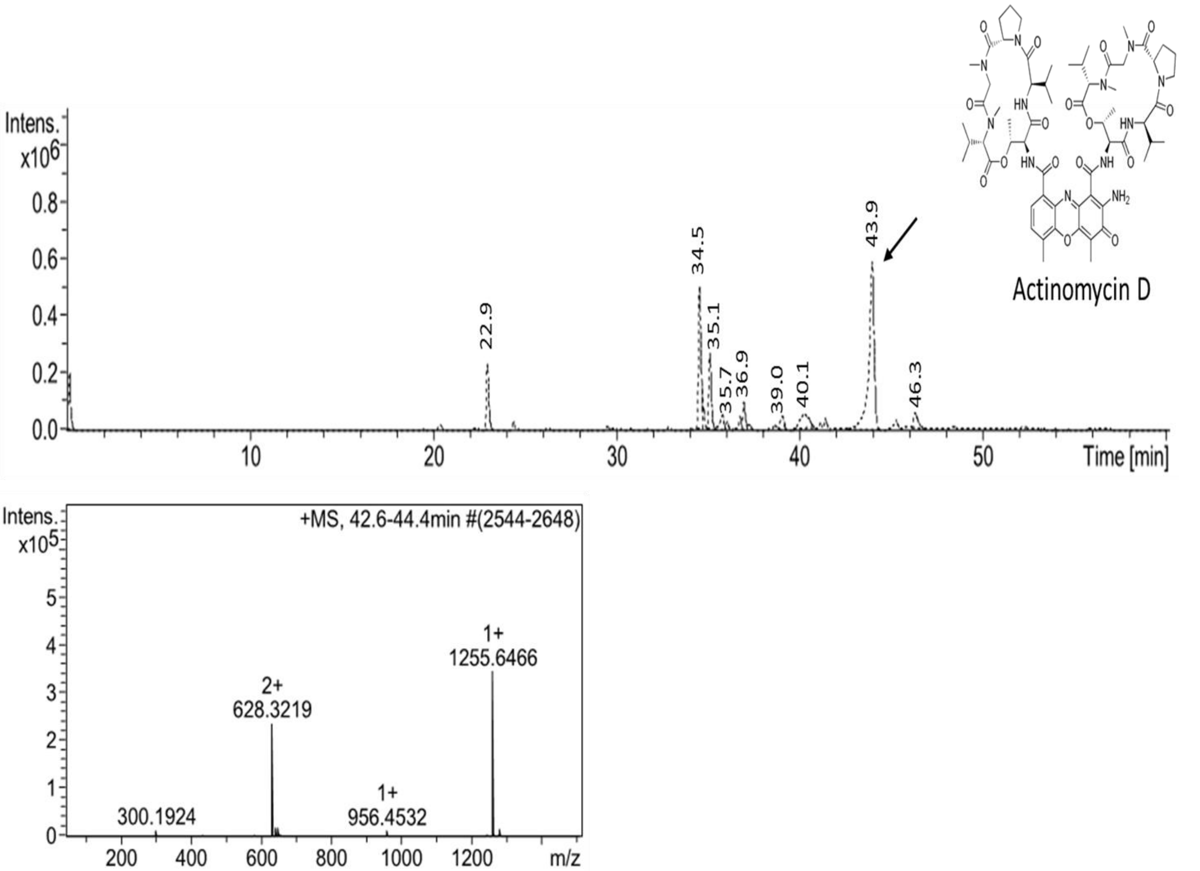

Table S1. Comparative analysis of housekeeping genes in *Streptomyces* sp. PJ85 and *S. actinomycinicus* RCU-197^T.

Four housekeeping genes including *gyr*B (DNA gyrase beta subunit), *rpo*A (RNA polymerase alpha subunit), *atp*D (ATP synthase subunit b), and *rpo*B (DNA-directed RNA polymerase subunit beta) of strain PJ85 were sequenced and submitted to the GenBank. These housekeeping genes of PJ85 and *S. actinomycinicus* RCU197^T were compared by using BLASTN programs available on National Center for Biotechnology Information (https://www.ncbi.nlm.nih.gov/). A > 97% identity of housekeeping sequences was the criterion used to define a potentially same species [51,52].

**Gene / GenBank Accession No.**	**Gene name**	**% identity**
*gyr*B / OP832340	DNA gyrase beta subunit	98.99
*rpo*A / OP832342	RNA polymerase alpha subunit	99.51
*atp*D / OP832339	ATP synthase subunit b	99.58
*rpo*B / OP832341	DNA-directed RNA polymerase subunit beta	99.08

References

51. Liu, Y.; Lai, Q.; Shao, Z. A Multilocus Sequence Analysis Scheme for Phylogeny of Thioclava Bacteria and Proposal of Two Novel Species. Front. Microbiol. 2017, 8, 1321. https://doi.org/10.3389/fmicb.2017.01321.

52. Ian, E.; Malko, D.B.; Sekurova, O.N.; Bredholt, H.; Ru, C.; Borisova, M.E.; Albersmeier, A.; Kalinowski, J.; Gelfand, M.S.; Zotchev, S.B. Genomics of Sponge-Associated Streptomyces spp. Closely Related to Streptomyces albus J1074: Insights into Marine Adaptation and Secondary Metabolite Biosynthesis Potential. PLoS. ONE 2014, 9, e96719. https://doi.org/10.1371/journal.pone.0096719.
